# Myco-Synthesized Molluscicidal and Larvicidal Selenium Nanoparticles: A New Strategy to Control *Biomphalaria alexandrina* Snails and Larvae of *Schistosoma mansoni* with an In Silico Study on Induced Oxidative Stress

**DOI:** 10.3390/jof8030262

**Published:** 2022-03-04

**Authors:** Mostafa Y. Morad, Heba El-Sayed, Ahmed A. Elhenawy, Shereen M. Korany, Abeer S. Aloufi, Amina M. Ibrahim

**Affiliations:** 1 Zoology and Entomology Department, Faculty of Science, Helwan University, Helwan 11795, Egypt; myame_mostafa@yahoo.com; 2 Botany and Microbiology Department, Faculty of Science, Helwan University, Helwan 11795, Egypt; drhebaelsayed39@gmail.com; 3 Chemistry Department, Faculty of Science, Al-Azhar University, Nasr City, Cairo 11884, Egypt; elhenawy_sci@hotmail.com; 4 Chemistry Department, Faculty of Science and Art, Al Baha University, Mukhwah, Al Baha 6531, Saudi Arabia; 5 Department of Biology, College of Science, Princess Nourah bint Abdulrahman University, P.O. Box 84428, Riyadh 11671, Saudi Arabia; smkorany@pnu.edu.sa; 6 Medical Malacology Department, Theodor Bilharz Research Institute, Giza 12411, Egypt; aminamd.ibrahim@yahoo.com

**Keywords:** *Penicillium chrysogenum*, *Biomphalaria alexandrina*, *Schistosoma mansoni*, selenium nanoparticles, molluscicide, larvicide, docking study

## Abstract

Schistosomiasis is a tropical disease with socioeconomic problems. The goal of this study was to determine the influence of myco-synthesized nano-selenium (SeNPs) as a molluscicide on *Biomphlaria alexandrina* snails, with the goal of reducing disease spread via non-toxic routes. In this study, *Penicillium chrysogenum* culture filtrate metabolites were used as a reductant for selenium ions to form nano-selenium. The SeNPs were characterized via UV-Vis spectrophotometer, Fourier transform infrared (FT-IR) spectroscopy, transmission electron microscopy (TEM), dynamic light scattering (DLS), and X-ray diffraction (XRD). Myco-synthesized SeNPs had a significant molluscicidal effect on *B. alexandrina* snails after 96 h of exposure at a concentration of 5.96 mg/L. SeNPs also had miracidicidal and cercaricidal properties against *S. mansoni*. Some alterations were observed in the hemocytes of snails exposed to SeNPs, including the formation of pseudopodia and an increasing number of granules. Furthermore, lipid peroxide, nitric oxide (NO), malondialdehyde (MDA), and glutathione s-transferase (GST) increased significantly in a dose-dependent manner, while superoxide dismutase (SOD) decreased. The comet assay revealed that myco-synthesized SeNPs could cause breaks in the DNA levels. In silico study revealed that SeNPs had promising antioxidant properties. In conclusion, myco-synthesized SeNPs have the potential to be used as molluscicides and larvicides.

## 1. Introduction

Schistosomiasis is a serious illness that has impacted the lives of people and animals around the world [1]. *Schistosoma mansoni* is a widely distributed parasitic species in many regions in Africa, the Middle East, and South America, where the intermediate host, a freshwater snail named *Biomphalaria* (phylum Mollusca, class Gastropoda), is located [2]. Snails have great medical, veterinary, and economic importance as they are the causative agent in transmitting diseases that affect many animals [3]. Until now, praziquantel has been widely used to treat adult trematode as well as cestode worms, but it is less effective against juvenile stages [4]. Thus, the urgent need for control strategies has evolved to control the snail population [5,6]. The chemical control of snail populations has many disadvantages, especially that it is very expensive, toxic to the non-target organism, and could accumulate in the environment [7]. Biological control of snail populations is a low-cost and effective alternative to chemical molluscicides [8].

Nanotechnology is an emerging technology that has been rapidly developed over the last two decades to enhance and manage a wide range of issues and problems in fields, such as health, food, the environment, agriculture, and numerous industries [9,10]. The emergence of nano-sized components (1–100 nm) is accomplished through three main methods: physical, chemical, and biological reactions [11]. Chemical and physical synthesis methods generally involve unique processing components and difficult conditions, such as hazardous chemicals, pressure, controlled pH and temperature, and large equipment. Moreover, these production techniques are expensive and produce undesired by-products that cause difficulties in the environment [12]. In contrast to chemical and physical procedures, the biological strategy is distinguished by its simplicity, speed, safety for the environment, and relatively inexpensive [13]. As a result, researchers are focusing on the biological approach, or green technique, of producing nanomaterials, which employs fungi, bacteria, yeast, algae, actinomycetes, and plants [14,15,16].

Fungi are one of the living organisms involved in the green synthesis of nanoparticles [17]. Furthermore, because of fungi’s versatility, high metal tolerance, ease of handling, high biomass output, and commercial feasibility, fungi are well-suited for the production of a wide range of nanoparticles [18]. *Penicillium chrysogenum* is one of the most common fungi that produces a large variety of metabolites, such as various enzymes, roquefortines, siderophores, fungisporin, penitric acid, indole-3-acetic acid, chrysogine, hydroxyemodin, and chrysogenin [19,20,21]. As a consequence, it could be used to fabricate a variety of metal and metal oxide nanoparticles.

Selenium is an essential nutrient that is necessary for good health and regulates a variety of cellular processes via selenium proteins [22]. Selenium is essential in the prevention of a range of diseases, including infectious diseases, hypercholesterolemia, cardiovascular disease, and some malignancies. SeNPs have significant antibacterial activity in naked [23,24] and conjugated forms, such as the selenium nanoparticles-lysozyme nanohybrid system [25]. Despite these numerous benefits, large doses of selenium can have negative side effects. As a result, reports are now focusing on the use of nanomaterials to avoid high doses of Se metal while retaining biological effects [26]. SeNPs have received a lot of recent interest due to their unique properties and biological activities. Selenium nanoparticles have shown biomedical and larvicidal effects, and they can be used for infection control [27]. The strategy of NPs’ toxicity at the cellular level has not been completely identified, but it may result in membrane disruption, protein oxidation, interruption of energy transduction, genotoxicity, the release of toxic constituents, and the formation of reactive oxygen species (ROS) [28]. The current work was conducted to test the use of myco-synthesized SeNPs by exposing *Penicillium chrysogenum* culture filtrate metabolites to sodium selenite as a tool for biological control against the intermediate host, *Biomphalaria alexandrina* snails, as well as larval stages of *Schistosoma mansoni*.

## 2. Materials and Methods

### 2.1. Isolation Conditions of the Fungal Isolate

The studied fungus was isolated from stones collected on the Mediterranean’s southern coast, at Alexandria, Egypt. Isolation was done on a medium comprising 10.0 g saja peptone, 3.0 g of yeast extract, 3.0 g of malt extract, 10.0 g of glucose, 30.0 g of NaCl, 25.0 g of agar, 1000 mL of distilled water, pH 7.5. One thousand microliters per litre of streptomycin was provided after the media had cooled. The culture was incubated for five days at 25 °C.

### 2.2. Molecular Identification

The fungal isolate was cultured in Czapek’s yeast extract agar medium (CYA) for 7 days at 28 °C [29]. The fungal mat was filtered through Whatman’s filter paper No. 1, and mycelial DNA was isolated using the Patho-gene-spin DNA/RNA extraction kit as per the manufacturer’s instructions (Intron Biotechnology Company, Korea). SolGent Company in Daejeon, South Korea, performed the polymerase chain reaction (PCR) and rRNA gene sequencing. ITS1 (forward) and ITS4 (reverse) primers were incorporated into the reaction mixture for PCR. ITS1 (5′-TCC GTA GGT GAA CCT GCG G-3′) and ITS4 (5′-TCC TCC GCT TAT TGA TAT GC-3′) are the two primers used. With the addition of ddNTPs to the reaction mixture, the PCR product was sequenced using the same primers [30]. The obtained sequences were analyzed using the National Center of Biotechnology Information’s (NCBI) website’s Basic Local Alignment Search Tool (BLAST).

### 2.3. SeNPs Myco-Synthesis by P. chrysogenum

The mycosynthesis of SeNPs was carried out according to Amin et al. [31] with some modifications. *P. chrysogenum* was cultivated on potato dextrose broth and incubated in a static incubator for 7 days at 25 °C. Mycelia were then separated from the culture supernatant by centrifugation (Sigma, 3-16PK, Osterode am Harz, Germany) at 10,000 rpm for 10 min [32]. After that, sodium selenite (Na_2_SeO_3_) was mixed with 15 mL of culture supernatant to obtain a final concentration of 3 mM, which was consequently incubated at 40 °C for 30 min until the formation of SeNPs. A change in the color of the culture supernatant from yellow to red confirmed the synthesis of SeNPs [33]. Finally, the biosynthesized SeNPs were centrifuged three times at 10,000 rpm for 10 min with double distilled water to purify them prior to being oven-dried at 60 °C for 48 h. A culture supernatant was used as a control under the same experimental conditions. The nanoparticles were kept in the refrigerator and re-dispersed with distilled water during use.

### 2.4. Characterization of the Myco-Synthesized SeNPs

A UV–Visible spectrophotometer, Zetasizer analyzer, X-ray diffraction instrument, transmission electron microscope (TEM), and Fourier transform infrared (FTIR) spectrophotometer were used to characterize the optical, morphological, structural, elemental, and functional characteristics of the synthesized SeNPs. The SeNPs absorbance was examined using a UV–visible spectrophotometer at wavelengths in the range of 400 to 800 nm (PerkinElmer Life and Analytical Sciences, CT, Ohio, USA). The average diameter size and distribution, as well as zeta potential charges, were determined by the particle size analyzer Dynamic Light Scattering (DLS) (Zetasizer Nano ZN, Malvern Panalytical Ltd., Malvern, UK) at a fixed angle of 173° at 25 °C. XRD was performed using a Bruker D8 DISCOVER Diffractometer, USA, with Cu-K radiation (λ = 1.54060 Angstrom) to determine the particles’ crystalline size. The relative intensity information was analyzed throughout a 2θ range of 5–100°. 2θ values and relative intensities (I/Io) were obtained from the chart, and core materials minerals were characterized using JCPDS carts. A high-resolution transmission electron microscope (HR-TEM; JEOL 2100, Japan) equipped with an electron diffraction pattern was also used to take transmission electron photographs. Fourier transform infrared spectroscopy was employed to investigate the elemental structure of SeNPs as well as the functional groups (FTIR; PerkinElmer, Ohio, USA). Triplicate samples were analyzed.

### 2.5. Investigation of Molluscicidal Activity of SeNPs

#### 2.5.1. Snails

The snails, *B. alexandrina* (Ehrenberg, 1831) (8–10 mm in diameter), were acclimatized in the Medical Malacology Laboratory, Theodor Bilharz Research Institute (TBRI), Giza, Egypt. Snails were housed in plastic aquaria (16 × 23 × 9 cm) and fed with oven-dried lettuce leaves, blue-green algae (*Nostoc muscorum*), and tetramin. The following properties were used in the experiment: dechlorinated aerated tap water (10 snails/L), pH: 7 ± 0.2, and temperature (25 ± 2 °C) were covered with glass plates. Thirty mg/L of calcium carbonate was added to the water to achieve its optimum hardness for snail fecundity, shell length, and growth [34].

#### 2.5.2. Molluscicidal Activity of SeNPs

A series of concentrations were prepared from the stock solution of SeNPs (95, 80, 65, 50, 35, and 25 mg/L) to calculate LC_90_ [35]. The snails (180) were subjected to a 96-h exposure period followed by a 24-h recovery period. Only dechlorinated water was used to keep the three control groups (30 snails) the same size. For each concentration, three replicates were used, each with ten snails. The mortality rate was measured and analyzed. [36].

#### 2.5.3. Miracidicidal and Cercaricidal Activity

As a control group, 10 mL of dechlorinated tap water was added to 102 newly formed miracidia or cercariae. To assess the effect of biosynthesized SeNPs on the newly formed miracidia or cercariae, about 5 mL of LC_10_ (31.826 mg/L) and LC_25_ (44.15 mg/L) of biosynthesized SeNPs were added to 102 newly formed miracidia or cercariae found in 5 mL of water [37]. Using the dissecting microscope, the vitality of miracidiae and cercariae was scored after 15, 30, 45, 60, 120, 180, 240, 300, and 360 min [38].

### 2.6. Experimental Design

Ten snails in each aquarium were exposed to the sub-lethal concentrations of SeNPs at LC_10_ (31.82 mg/L) or LC_25_ (44.15 mg/L) for 96 h (exposure), followed by another 24 h of recovery, two weeks of repeating, followed by two weeks of recovery.

#### 2.6.1. Hemolymph and Light Microscopy Preparation

The hemolymph was withdrawn from the heart using a capillary tube [39]. Part of the collected hemolymph was put on a glass slide, making a monolayer of hemocytes. Then the slides were dried in a moist chamber for 15 min at room temperature, followed by 5 min of dehydration in methanol, and finally stained for 20 min with 10% Giemsa stain (Aldrich) [40].

#### 2.6.2. Comet Assay

After the exposure of *B. alexandrina* snails (8–10 mm) to SeNPs at concentrations of LC_10_ (31.28 mg/L) and LC_25_ (44.15 mg/L) for 96 h, the head feet of 10 snails from each group were cut and kept at −80 °C until they were needed. The single-cell gel assay was used to measure the comet assay for the detection of DNA breaks, as described by Grazeffe et al. [41] and Ibrahim and Sayed [42]. The slides were coded independently and scored independently.

#### 2.6.3. Tissue Preparation

The soft tissues of the exposed and control groups were obtained by crushing the shells of the snails using two slides, weighing (1 g tissue/10 mL of phosphate buffer), and homogenizing with a glass Dounce homogenizer. Then, the tissue homogenates were centrifuged (Sigma, 3-16PK, Osterode am Harz, Germany) at 3000 rpm for 10 min, and the supernatants were stored at −80 °C until used.

##### Determination of Testosterone (T) and Estradiol (E2) Hormones Concentrations

The hormonal activity of T and E2 was estimated following the manufacturer’s instructions, in which T concentrations were measured using an EIA kit (Abia Testosterone, REF, DK. 040.01.3), while for E2 concentration, an immunoassay test kit (BioCheck, Inc., South San Francisco, CA94080, USA) was used [42].

##### Investigation of the Antioxidant Responses: Superoxide Dismutase (SOD); Glutathione S-Transferase (GST), Nitric Oxide (NO), Malondialdehyde (MDA), and Total Antioxidant Capacity (TAC)

For each group, biochemical changes in the tissue homogenate’s supernatant were monitored. Biodiagnostic kits (Biodiagnostic, Dokki, Giza, Egypt) were used to assess SOD [43]. In addition, cell MDA (lipid peroxide) was measured using the Ohkawa et al. [44] method, and GST was detected using the Beutler [45] method. TAC was determined with the kit (Cat. No. TA 2513) [46]. According to Bellos et al. [47], NO was estimated.

### 2.7. The Molecular Docking Study

The inhibitory potential of Na_2_SeO_3_ was investigated using two enzymes from the cellular anti-oxidant mechanism, SOD and GST. Three-dimensional crystals of human SOD 1 (PDB id: 5YTU) and SOD 2 (PDB id: 13GS) complexed with isoproterenol and sulfasalazine, respectively. These catalases were obtained from (www.rcsb.org/, 10 February 2022) in.pdb format. Na_2_SeO_3_ and reference inhibitors (isoproterenol (for SOD) and sulfasalazine (for GST) were selected. (www.pubchem.ncbi.nm.nih.gov/, 10 February 2022) was used for generating the 3D structures for ligands in .sdf format. MOE2015 is advanced computational modelling software for evaluating ligand → active site interactions. MOE 2015 conducted a docking experiment, which was used to correct errors in active sites during the structure preparation reaction. Hydrogens were added after the correction, and partial charges (AMBER12: EHT) were calculated. Energy minimization was carried out (AMBER12: EHT, root mean square gradient: 0.100). The MOE Site Finder program, which uses a geometric approach to calculate putative binding sites in a protein starting from its tridimensional structure, was used to find the receptor’s binding site. This method is based on alpha spheres, which are a generalization of convex hulls, rather than energy models. The binding sites predicted by the MOE Site Finder module in the holo-forms of the investigated proteins confirmed the binding sites defined by the co-crystallized ligands.

### 2.8. Statistical Analysis

The Probit facility analyzed the median lethal and lethal concentration values [48]. The mean values of the experimental and control groups were compared using the Student’s *t*-test [49]. The data was analyzed using the statistical software SPSS version 20 for Windows (SPSS, Inc., Chicago, IL, USA). The results were expressed as the average value ± S.E.

## 3. Results

### 3.1. Molecular Identification of the Fungal Strain

**Figure 1:**
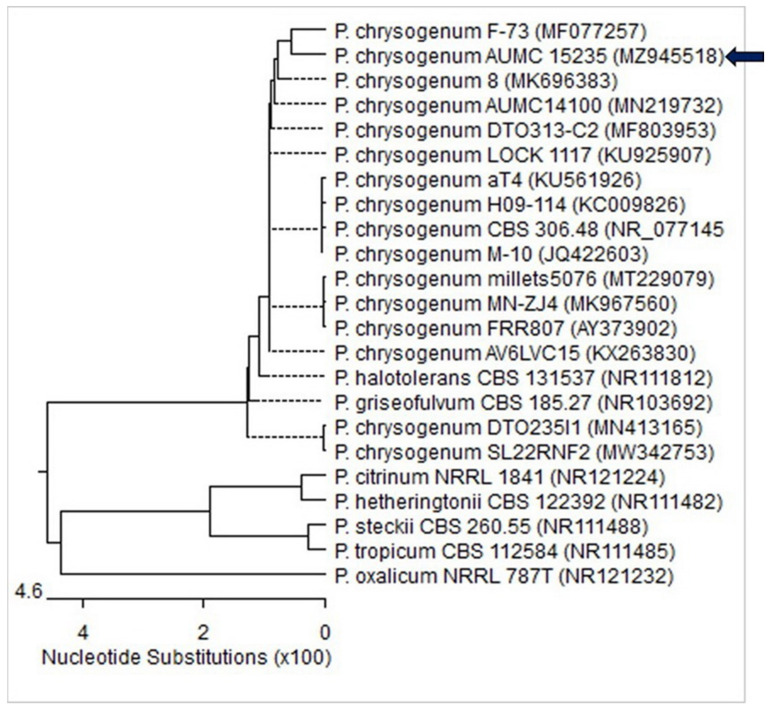
Phylogenetic tree of the fungal isolate depending on ITS sequence (GenBank accession no. MZ945518, arrowed) aligned with closely similar strains in the GenBank. (*P. = Penicillium*). The sequences were phylogenetically analyzed utilizing MegAlign (DNA Star) software version 5.05.

The ITS sequences of the fungal isolate’s rDNA were aligned with strains from GenBank that were genetically related. It demonstrated 99–100% identity and 100% coverage with several *Penicillium chrysogenum* strains, including the type strain CBS 306.48 (GB no.: NR 077145). The sequence was registered in the GenBank database under the accession number MZ945518 (Figure 1).

### 3.2. The Myco-Synthesis of SeNPs by P. chrysogenum

**Figure 2:**
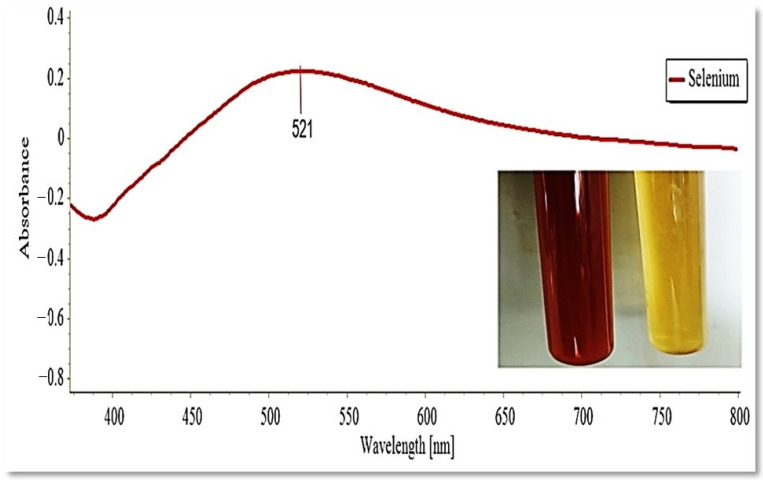
UV–Visible absorption spectrum and brick-red color of the myco-synthesized selenium nanoparticle.

After 30 min of incubation, the color of the culture medium changed from yellow to brick-red after the culture filtrate was treated with 1 mM Na_2_SeO_3_ (Figure 2). Following incubation, the presence of a red-brick color inside the culture medium was clear evidence that the extracellular metabolites rapidly reduced selenite ions to the elemental Se (Se0) form [33]. The SeNPs’ productivity was calculated to be around 38 mg/100 mL.

### 3.3. Characterization of the Myco-Synthesized SeNPs

UV–Visible spectrophotometry was used to monitor the SeNPs production in the culture filtrate, revealing a strong and broad surface plasmon resonance (SPR) peak at 521 nm, which is an SeNPs feature (Figure 2). In the control, however, no absorption peak corresponding to the SeNPs was found.

**Figure 3:**
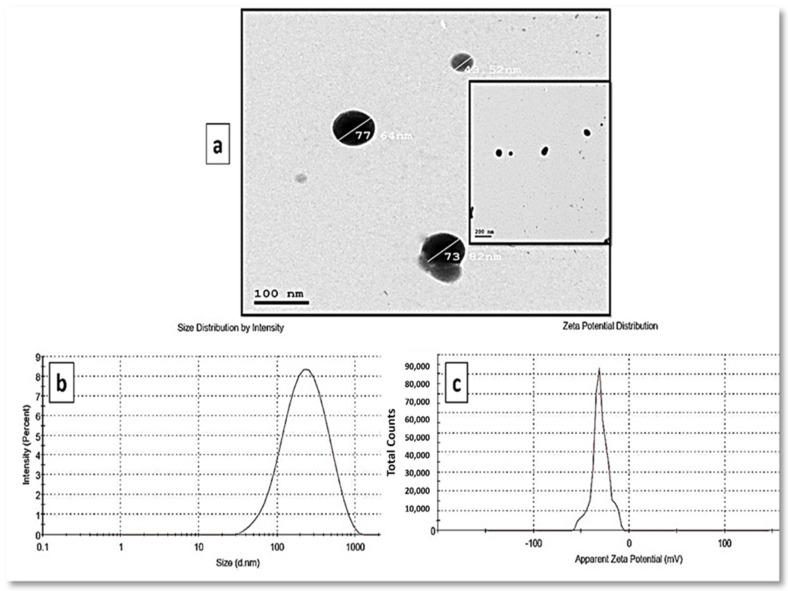
Morphological characterization of myco-synthesized SeNPs (**a**) TEM photographs of myco-synthesized selenium nano-selenium using *P. chrysogenum* culture filtrate at the scale of 100 nm and 200 nm, (**b**) size distribution pattern, and (**c**) zeta potential distribution.

The synthesis of polydispersed spherical SeNPs with diameter sizes ranging from 44 to 78 nm was revealed by TEM analysis of a colloidal solution of myco-synthesized SeNPs (Figure 3a). Figure 3b shows the average size distribution in the SeNP solution as determined by DLS. According to the results obtained, the myco-synthesized SeNPs were measured to have an average diameter of 207 nm. DLS determines the size of SeNPs, which is influenced by biomolecules coated on their surfaces as stabilizers as well as their metallic cores. The stability of SeNPs was assessed using the zeta potential assessment of particle surface charge, which revealed a mean zeta potential of −32.4 mV (Figure 3c).

**Figure 4:**
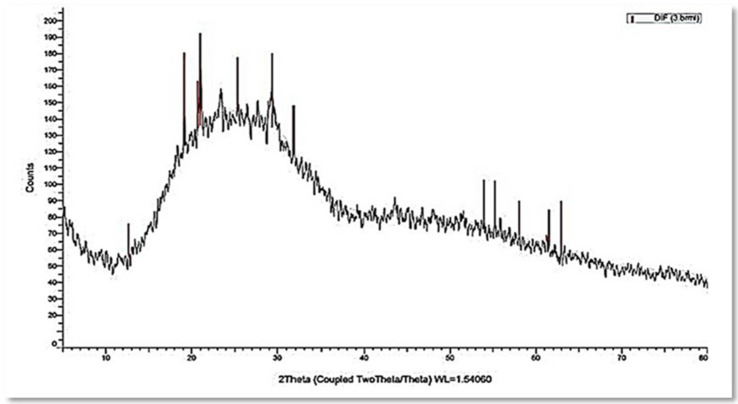
XRD spectrum of myco-synthesized selenium nanospheres.

The XRD results revealed a broad pattern with no clear Bragg peaks. While there were no significant peaks, smaller peaks at 2Ѳ values were found at 12.658°, 19.146°, 20.712°, 21.011°, 25.352°, 29.402°, 31.830°, 53.938°, 55.218°, 58.039°, and 61.540°. The results demonstrated that myco-synthesized SeNPs are rather more amorphous than crystalline (Figure 4).

**Figure 5:**
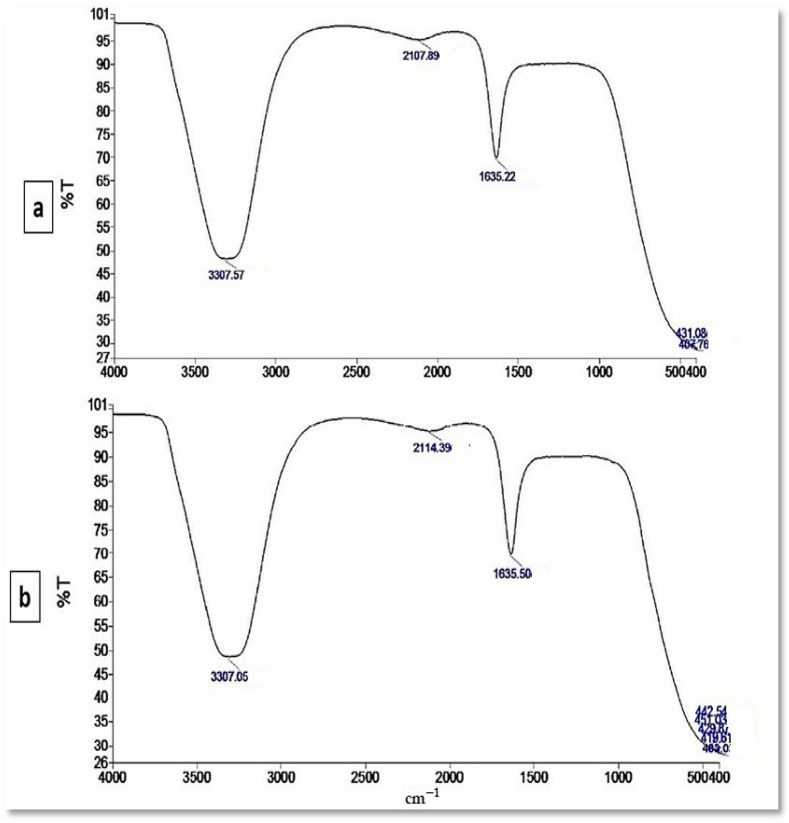
FTIR pattern (**a**) culture supernatant, and (**b**) myco-synthesized selenium nanoparticles, where *Y*-axis represented the transmission (%T) and *X*-axis represented the wavenumber (cm^−1^).

The presence of various functional groups in metabolites that are responsible for SeNP myco-synthesis, capping, and stabilization was determined using FTIR measurements. The FTIR for the culture supernatant of *P. chrysogenum* was analyzed and showed five intense peaks observed at 3307.57, 2107.89, 1635.22, 431.08, and 407.76 cm^−1^ (Figure 5a). These peaks were shifted to eight peaks in the chart of SeNPs. The interaction of metabolites with SeNPs is illustrated by wavenumbers at 3307.05, 2114.39, 1635.50, 451.03, 442.54, 429.87, 419.61, and 403.05 cm^−1^ (Figure 5b).

### 3.4. Effects of Selenium Nanoparticles against B. alexandrina Snails

**Figure 6:**
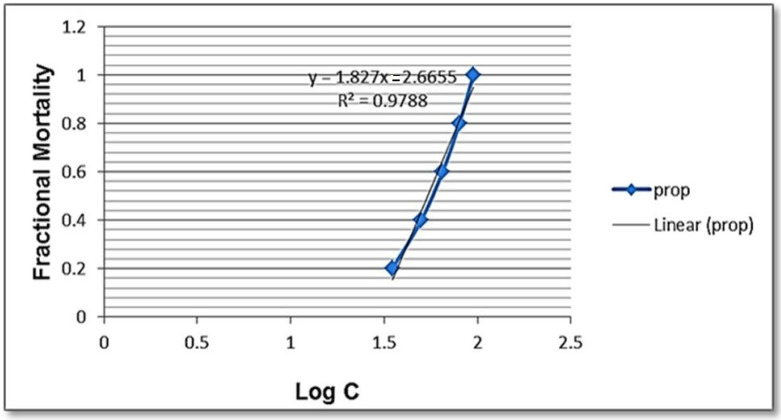
Molluscicidal effect of SeNPs against *B. alexandrina* snails as shown in probit analysis.

The present findings revealed that SeNPs have a molluscicidal effect against adult *B. alexandrina* snails after 96 h of exposure at LC_50_ 5.96 mg/L (Table 1 and Figure 6).

**Figure 7:**
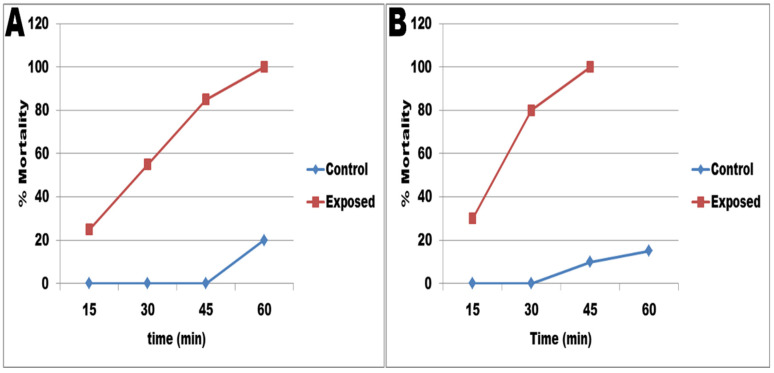
The miracidicidal (**A**) and cercaricidal (**B**) activities of SeNPs.

The current results showed that SeNPs have a toxic effect on *S. mansoni* stages, as shown in Figure 7. All miracidae exposed to SeNPs died after 60 min, compared to only 20% of the deaths in the control group (Figure 7A), while 45 min was enough to kill all exposed cercariae, compared to 10% of the control group.

**Figure 8:**
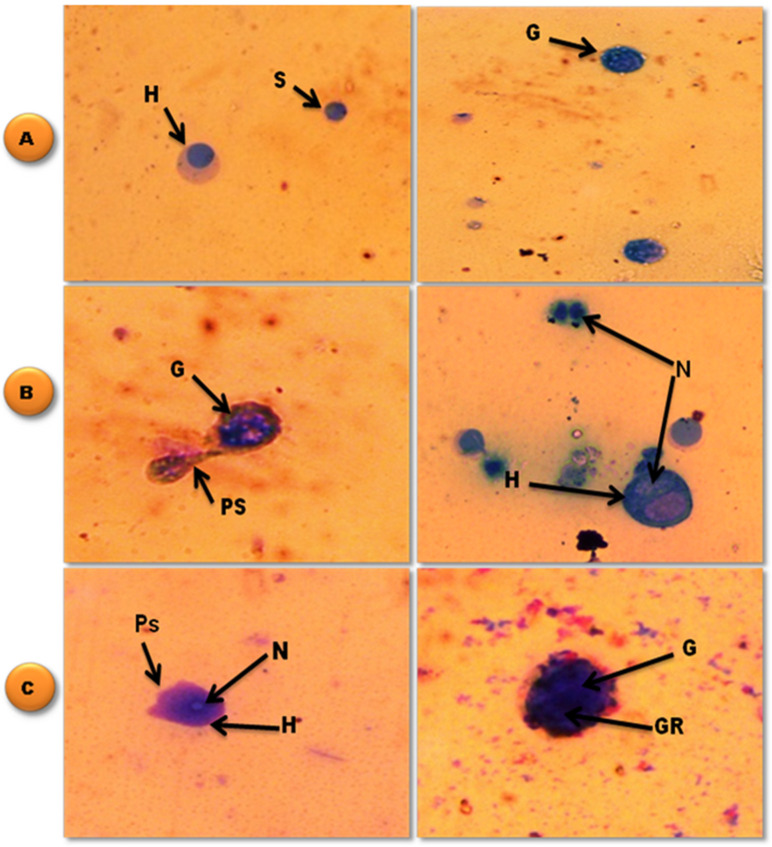
Photomicrographs (×40) of different types of hemocytes.(**A**) hemocytes of control group of adult *B. alexandrina* snails, (**B**) *B. alexandrina* snails hemocytes after exposure to LC_10_ of SeNPs (31.826 mg/L), (**C**) hemocytes of adult *B. alexandrina* snails after exposure to LC_25_ of SeNPs (44.15 mg/L). Abbreviations, G: granulocyte, GR: granules, H: hyalinocyte, N: nucleus, PS: pseudopodia, S: small round.

The examination of hemocyte monolayers by light microscope showed the presence of three cell types of hemocytes in the control group: small hemocytes, hyalin oocytes, and granulocytes (Figure 8A). Hemocytes of exposed *B. alexandrina* snails suffered from many changes. The exposure to LC_10_ (31.826 mg/L) of SeNPs showed numerous granules and newly formed pseudopodia in the granulocytes, while hyalinocytes suffered from incomplete cell division where the nucleus divided, forming two nuclei without cell membrane separation (Figure 8B). The exposure to LC_25_ (44.15 mg/L) of SeNPs resulted in increased observed granules with an irregular cell membrane in granulocytes, while newly small pseudopodia were seen in hyalinocytes (Figure 8C).

**Figure 9:**
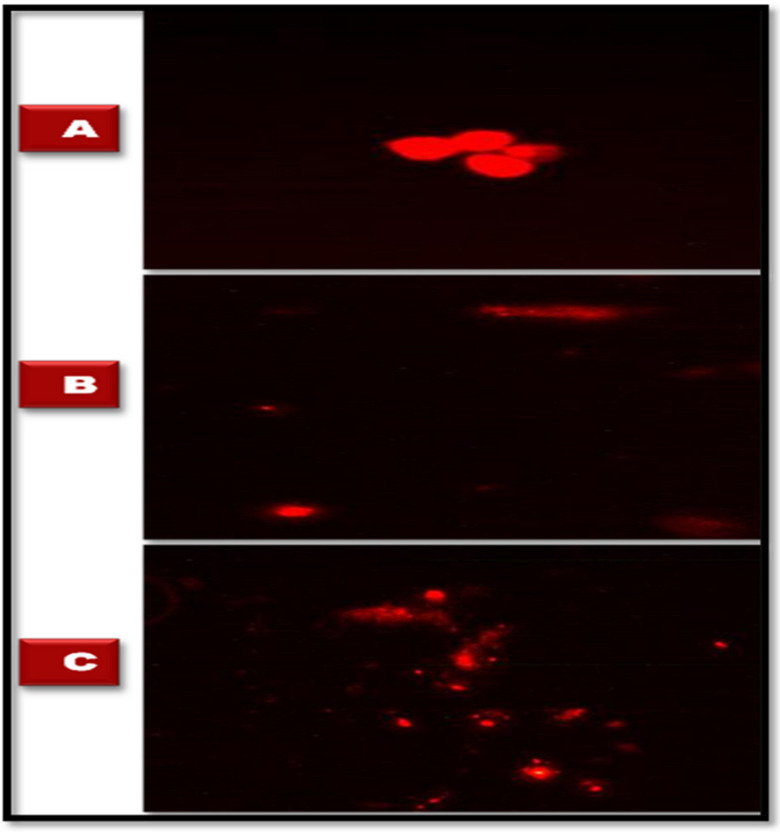
Ranks of comet according to the percent of DNA in the tail. (**A**) Control group, (**B**) *B. alexandrina* snails after exposure to LC_10_ of SeNPs (31.826 mg/L), (**C**) adult *B. alexandrina* snails after exposure to LC_25_ of SeNPs (44.15 mg/L).

The current study found that after the exposure to sublethal concentrations of SeNPs, there were DNA breaks where the percentage of the comet, tail length, percent DNA in tail, and tail moment were increased (*p*< 0.05 and 0.01) compared to control snails (Table 2 and Figure 9).

The current findings revealed that testosterone (T) and estradiol (E2) levels were significantly higher (*p* < 0.05) after exposure to sublethal concentrations when compared to the control group (Table 3).

Significant increases in a concentration-dependent manner (*p* < 0.05) of MDA, NO, and GST were noticed after in vivo exposure of *B. alexandrina* snails to sub-lethal concentrations of SeNPs. On the other hand, SOD activity was significantly decreased (*p* < 0.05) while TAC was insignificantly decreased (Table 4).

### 3.5. Molecular Docking Study

**Figure 10:**
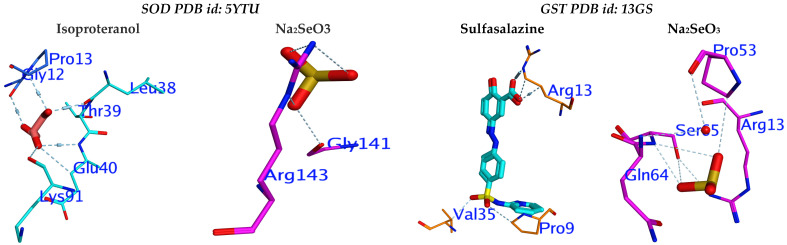

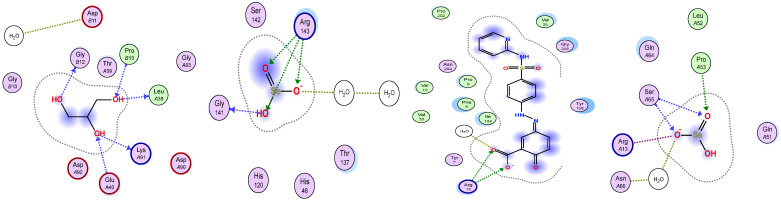
2D and 3D docked interaction map for the Na_2_SeO_3_ compounds into the active site of SOD and GST.

The two enzymes, SOD and GST, were selected for the cellular antioxidant mechanism to determine the inhibitory potential of sodium selenite. Three-dimensional structures of human SOD 1 complexed with isoproterenol (PDB id: 5YTU) and human GST complexed with sulfasalazine (PDB id: 13GS) were used for molecular docking. The tested compound revealed high efficiency against the respective receptor binding sites of SOD and GST (Figure 10). The molecular docking showed that Na_2_SeO_3_ succeeded in binding to similar active sites in the same spot as the original inhibitor, which suggests that Na_2_SeO_3_ blocks the receptors in the same way.

The inhibition activities for the enzymes were examined using interaction-free energy, which is known as the docking score. Na_2_SeO_3_ showed promising docking and H-interaction scores (−5.03 and −4.70 Kcal/mol), respectively, against SOD and GST, and its compound displayed promising scores (Table 5).

## 4. Discussion

The application of green synthesis nanoparticles for freshwater snail control is novel. In this approach, natural metabolites extracted from plants and microorganisms are used to synthesize nanoparticles. Natural molecules are thought to produce safer nanoparticles than the more toxic chemical polymers used in the fabrication process [50]. The activity of metabolites released by various organisms, including plants, fungi, actinomycetes, bacteria, and algae, were exploited in metal ion reduction, capping, and stability for the biosynthesis of metal nanoparticles [18]. In the current study, the reducing capacity of the culture filtrate metabolites of *P. chrysogenum* was used to synthesize SeNPs. *P. chrysogenum* is capable of producing a diverse spectrum of secondary biomolecules that work as a biocatalyst to reduce and stabilize nanoparticles. Proteins, several enzymes, and carbohydrates are some of these molecules [10,51]. Joshi et al. [52] observed that the fungal strain’s ability to reduce Se ion and synthesize SeNPs is due to the numerous extracellular proteins and enzymes. Amin et al. [31] reported that *Penicillium chrysogenum F9* was employed as a biocatalyst for SeNPs biosynthesis. The resultant color shifting was caused by the surface plasmon resonance of monoclinic Se particles [53]. The production of SeNPs in culture filtrate was measured using UV–Visible spectroscopy, which revealed a strong and broad peak at 521 nm. It was reported that the absorbance values of SeNPs were determined at 300 nm and another value at 540 nm [54,55]. SeNPs displayed absorbance at approximately 520 nm due to Mie scattering [56]. Ullah et al. [57] stated that the maximum absorption peak of selenium nanoparticles synthesized by *Bacillus subtilis* BSN313 was at 650 nm. The current findings were consistent with those of Ranjitha and Ravishankar [58], who indicated that selenium nanoparticles synthesized by *Streptomyces griseoruber* had a maximum peak at 575 nm. The sizes determined by TEM (44 to 78 nm) and DLS (207 nm) differed because DLS examines the hydrodynamic quantity while TEM examines the solid core [59]. The negative charge of particles indicates the electrostatic stability of the synthesized nanoparticles as reported by [32]. The electrostatic stability of the synthesized colloidal nanoparticle solution is indicated by a zeta potential greater than +30 mV or less than −30 mV [60]. The XRD results revealed that the myco-synthesized SeNPs are rather more amorphous than crystalline. This amorphous nature is consistent with previous research with lycopene [61], *Pseudomonas stutzeri* [62], and *Withania somnifera* [63]. FTIR analysis revealed the interaction between *P. chrysogenum* metabolites and SeNPs. The signal band at 3307.05 cm^−1^ corresponds to N–H, C–H, and O–H stretching vibrations, suggesting the presence of primary amine in the fungal proteins [31], alkyne, and alcohol, respectively [64]. This demonstrates the importance of N–H-containing proteins in the reduction of Se ions and the formation of SeNPs. The band at 2114.39 cm^−1^ corresponds to the presence of alkyne. Furthermore, the peak at 1635.50 cm^−1^ was associated with various peptide linkage and polysaccharide ring moieties such as N-H, C=N, C=O, and C=C [33]. The bands at 451.03, 442.54, 429.87, 419.61, and 403.05 cm^−1^ revealed the binding of SeNPs with the metabolites of *P. chrysogenum* culture filtrate. According to these results, many functional groups of organic compounds, such as proteins and polysaccharides, present in the culture filtrate of *P. chrysogenum* are involved in the capping, stability, and reduction of SeNPs.

The present study revealed that SeNPs have molluscicidal activity against *B. alexandrina* after 96 h of exposure. These findings are consistent with those of Osman et al. [65], who observed that *B. alexandrina* snails treated with *Aspergillus fumigatus* fungal extract showed a high molluscicidal effect. In addition, Abdel-Hamid and Mekawey [65] reported that the myco-biosynthesis of silver nanoparticles (AgNps) from the two fungi, *Paecilomyces variotii* and *Aspergillus niger*, has molluscicidal activity against *B. alexandrina* snails.

Moreover, the current research found that SeNPs had miracidicidal and cercaricidal properties. Many similar studies have revealed the effective role of SeNPs against many other parasitic species. Mahmoudvand et al. [66] showed that Se NPs could kill promastigote and amastigote stages of *Leishmania major*. Similarly, Alkhudhayri et al. [67] found that Se NPs have anti-coccidial, anti-apoptotic, and anti-inflammatory effects against the Eimeria parasite in the jejunum of mice. Furthermore, Se nanoparticles showed promising protective roles against mice infected with *Schistosoma mansoni* [68]. Comparing the Se nanoparticles’ activity against meracidia and cercariae, the present results showed a faster mortality rate of cercariae than the meracidial rate. Unlike the present study, Osman et al. [65] showed that the fungal extract of *Aspergillus fumigatus* caused a higher mortality rate of miracidia than that of cercariae after exposure to the same experimental period. The mortality rates of both larval stages appear to differ depending on their biological nature and their internal structure [69].

One of the most sensitive tools to detect DNA defects is the comet assay, which detects DNA single-strand breaks [70]. The present results of the comet assay revealed that SeNPs induced DNA damage in *B. alexandrina*. Similarly, Ali [71] found that TiO_2_NP induced DNA damage in the freshwater snail *Lymnea leuteola*. Moreover, the author reasoned that these DNA breaks might be related to the oxidative stress that might be generated after the treatment. In good accordance with the present results, Ibrahim and Ghoname [72] found that exposure of *B. alexandrina* snails to the leaves of *Anagalis arvensis* aqueous extracts caused DNA breaks revealed by the comet assay. Inline supporting these results, Wang et al. [73] named the process of metal oxide NP transport ions onto the cells as “Trojan-horse type carriers”, causing serious damage of metal oxide NPs affecting the DNA molecules.

In gastropods, hemocytes, found in the hemolymph, represent the main component of the immune response [74]. According to their morphology, Cavalcanti et al. [75] reported three different types of haemocytes in *B. alexandrina*; spherical, small (undifferentiated), hyalinocytes, and granulocytes. The current study revealed that after exposure to LC_10_ (31.82 mgL^−1^) of SeNPs, the granulocytes showed pseudopodia and hyalinocytes had incomplete cell division, At exposure to 44.15 mg/L, some granulocytes formed many granules with an irregular cell membrane, and some hyalinocytes formed pseudopodia. These results are similar to those obtained by Abdel-Hamid and Mekawey [76], who found that hemocytes of treated *B. alexandrina* with LC_25_ of both *P. variotii* and *A. niger* AgNPs showed many alterations in their morphology, such as apoptotic hemolymph cells and fragmented, vacuolated, and degenerated cytoplasm. In agreement with the current study, Ibrahim et al. [77] exposed *B. alexandrina* to butralin, glyphosate isopropyl ammonium, or pendimethalin herbicides, and they observed that many granules and pseudopodia were produced by granulocytes while the hyalinocytes revealed a shrunken nucleus. Ray et al. [78] reported that hemocytes are the main immune cells working by phagocyting foreign particles in Mollusca species. Donaghy et al. [79] suggested that the observed pseudopodia of granulocytes may be a method of phagocytosis used to eliminate these foreign particles.

In the current study, the exposure of *B. alexandrina* to SeNPs induced oxidative stress that was noticed in the high levels of MDA in a concentration-dependent manner. Ohkawa et al. [44] reported that MDA is the most catastrophic effect of ROS, which is formed by the peroxidation of lipid membranes. Like the current study, Khalil [80] reported that MDA was significantly increased in the snail *Lanistes carinatus* after exposure to chlorpyrifos. These findings are similar to those of Ibrahim and Sayed [42], who found that malondialdehyde (MDA) increased in a concentration-dependent manner after the treatment of *B. alexandrina* snails with sub-lethal doses of oxyfluorfen herbicide (LC_0_, LC_10_, or LC_25_).

The antioxidant enzymes SOD and GST have a pivotal role in the elimination of ROS and modulate the response of living organisms to oxidative conditions. GST levels increased significantly after LC_25_ exposure, whereas SOD levels decreased significantly after LC_10_ and LC_25_ exposure. These results are consistent with the observations obtained by Khalil [80], who found that the GST activity was significantly increased in the adult freshwater snail *L. carinatus* when treated with chlorpyrifos. Similarly, the levels of SOD decreased in the snail *B. alexandrina* after treatment with atrazine and Roundup [81]. In contrast, Ibrahim and Sayed [42] found that the activity of SOD increased after the exposure of *B. alexandrina* to sub-lethal concentrations of oxyfluorfen herbicide. The unexpected suppression of SOD might result from protein degradation through oxidative damage to SOD or gene expression modifications [82]. One of the most effective innate immune defence mechanisms is the production of nitric oxide, which leads to the cytotoxicity of the invading pathogens in mollusks [78]. In the present study, the NO concentration significantly increased after the exposure to sublethal LC_10_ and LC_25_ of SeNPs. These results are in agreement with the findings of Wang et al. [83] who reported that the exposure of snail *B. straminea* to pyridyl phenyl urea derivatives led to high activity of NO. Also, Saleh et al. [84] found increasing NO activity after the treatment of *B. alexandrina* with the veterinary antibiotics oxytetracycline and trimethoprim-sulphadiazine.

The molecular docking method for examining receptor-ligand interactions is an important tool for predicting the inhibition actions of the enzymes associated with antioxidant activity. MOE 2016 [85] was used to perform the docking experiment. The enzymatic components include (SOD; PDB id: 5YTU [86]) and (GST; PDB id: 13GS [87]), the most potent non-enzymatic cellular antioxidant that is used by GST and GPx to neutralize oxidants [88]. SOD catalyzes the transformation of (O_2_^.−)^ → (H_2_O_2_), which is responsible for reducing the RONS levels [89]. The docking study aimed to determine the potential of Na_2_SeO_3_ in altering the cellular antioxidant defence system using molecular modeling. The observed results suggested the inhibition potency of Na_2_SeO_3_ against SOD and GST by interfering with their active important amino acids for catalytic sites.

The physiological and genotoxicological properties of *Biomphalaria alexandrina* snails were negatively impacted by myco-synthesized SeNPs. However, more research should be conducted to determine their effects on freshwater zooplanktonic species, such as the water flea *Daphnia magna*, which is used as a non-target organism for toxicity assessment in aquatic ecosystems found in the same habitat as *B*. *alexandrina* [90].

## 5. Conclusions

Myco-synthesized SeNPs have molluscicidal activity against the snail *Biomphalaria alexandrina* and larvicidal activity against *Schistosoma mansoni* larval stages, which could lead to a decrease in the spread of schistosomiasis. Further studies, including the effects on other (non-target) organisms and sub-lethal effects on snail fecundity/and fertility, are needed to determine whether the myco-synthesized SeNPs can be used as a promising tool for biological control and to replace the toxic synthetic chemical molluscicides.

## Figures and Tables

**Table 1:** Myco-synthesized SeNPs’ molluscicidal activity against *B. alexandrina* snails after 96 h of exposure.

Lethal Concentration Doses	LC_10_	LC_25_	LC_50_	LC_90_	Slope
**Concentration (mg/L)**	31.826	44.15	57.85	83.87	1.4

**Table 2:** DNA breaks after SeNPs exposure to *B. alexandrina* snails.

	Comet %	Tail Length (px)	% DNA in Tail	Tail Moment
**Control**	14.95	8.15 ± 0.42	23.79 ± 3.75	1.955 ± 0.402
**LC_10_**	15.85	10.507 ± 0.54 *	33.66 ± 1.43 *	3.529 ± 0.03 *
**LC_25_**	19.5	11.96 ± 1.37 *	33.588 ± 5.7 *	4.094 ± 1.15 *

1 px = 0.24 µm; * = significant compared to control at *p* < 0.05.

**Table 3:** SeNP exposure effects on (T) and (E2) concentration of *B. alexandrina* snails.

Groups	Testosterone (nmol/L)	Estradiol (pg/mL)
Control	20 ± 0.52	100 ± 3.1
LC_10_	30 ± 0.71 *	300 ± 5.2 *
LC_25_	35 ± 0.56 *	1000 ± 4.6 *

* = significant compared to control at *p* < 0.05.

**Table 4:** Effects of SeNP exposure on some biochemical parameters of *B. alexandrina* snails.

Biochemical Parameters	MDA (nmol/g.tissue)	NO (µmol/L)	SOD (U/g.tissue)	GST (U/g.tissue)	TAC (mM/L)
Control	9.811 ± 0.012	136.75 ± 0.42	8.36 ± 0.1	0.85 ± 0.05	1.505 ± 0.4
LC_10_	10.612 ± 0.4 *	148 ± 0.34 *	5.045 ± 0.02 *	0.927 ± 0.1	1.488 ± 0.21
LC_25_	14.47 ± 0.3 *	322 ± 0.21 *	3.624 ± 0.2 *	1.0545 ± 0.03 *	1.375 ± 0.3

* = significant compared to control at *p* < 0.05.

**Table 5:** The docking energy scores (kcal/mol) for Na_2_SeO_3_.

	ΔG	rmsd	E.vdw	E.Int	*E._H.B._*	Eele
5YTU	−5.03	3.86	−266.94	−1.86	−11.10	−27.82
13GS	−4.70	1.53	−266.52	−2.18	−10.09	−27.31

ΔG: The ligand’s free binding energy from a given conformer, E.Int.: affinity binding energy of hydrogen bond interaction with the receptor; ***E._H.B._***: hydrogen bonding energy between protein and ligand; Eele: electrostatic interaction with the receptor; Evdw: Van der Waals energies between the ligand and the receptor.

## Data Availability

All data generated or analyzed during this study are included in this article.

## References

[1] (2020). WHO Schistosomiasis. https://www.who.int/news-room/fact-sheets/detail/schistosomiasis.

[2] Ibrahim A.M., Sayed S.S.M. (2021). Assessment of the molluscicidal activity of the methanolic seed extracts of Ziziphus spina-christi and Carica papaya on immunological and molecular aspects of *Biomphalaria alexandrina* snails. Aquac. Res..

[3] Loker E.S., Mkoji G.M., Secor W.E., Colley D.G. (2005). Schistosomes and Their Snail Hosts BT—Schistosomiasis. Schistosomiasis.

[4] Faust C.L., Crotti M., Moses A., Oguttu D., Wamboko A., Adriko M., Adekanle E.K., Kabatereine N., Tukahebwa E.M., Norton A.J. (2019). Two-year longitudinal survey reveals high genetic diversity of *Schistosoma mansoni* with adult worms surviving praziquantel treatment at the start of mass drug administration in Uganda. Parasites Vectors.

[5] Abdel-Tawab H., Ibrahim A.M., Hussein T., Mohamed F. (2021). Mechanism of action and toxicological evaluation of engineered layered double hydroxide nanomaterials in *Biomphalaria alexandrina* snails. Environ. Sci. Pollut. Res..

[6] Ibrahim A.M., Bakry F.A. (2019). Assessment of the molluscicidal impact of extracted chlorophyllin on some biochemical parameters in the nervous tissue and histological changes in *Biomphalaria alexandrina* and *Lymnaea natalensis* snails. Invertebr. Neurosci..

[7] Elsareh F., Abdalla R., Abdalla E. (2016). The effect of aqueous leaves extract of *Solenostemma argel* (Del Hayne) on egg masses and neonates of *Biomphalaria pfeifferi* snails. J. Med. Plants.

[8] Abd El Ghaffar M.M., Sadek G.S., Harba N.M., Abd El Samee M.F. (2018). Evaluation of the effect of some plant molluscicides on the infectivity of *Schistosoma mansoni* cercariae. Menoufia Med. J..

[9] Rani P., Kaur G., Rao K.V., Singh J., Rawat M. (2020). Impact of Green Synthesized Metal Oxide Nanoparticles on Seed Germination and Seedling Growth of *Vigna radiata* (Mung Bean) and *Cajanus cajan* (Red Gram). J. Inorg. Organomet. Polym. Mater..

[10] Fouda A., Abdel-Maksoud G., Abdel-Rahman M.A., Salem S.S., Hassan S.E.D., El-Sadany M.A.H. (2019). Eco-friendly approach utilizing green synthesized nanoparticles for paper conservation against microbes involved in biodeterioration of archaeological manuscript. Int. Biodeterior. Biodegrad..

[11] Pham D.T.N., Khan F., Phan T.T.V., Park S.-k., Manivasagan P., Oh J., Kim Y.M. (2019). Biofilm inhibition, modulation of virulence and motility properties by FeOOH nanoparticle in *Pseudomonas aeruginosa*. Braz. J. Microbiol..

[12] Raliya R., Tarafdar J.C., Choudhary K., Mal P., Raturi A., Gautam R.K.S., Singh S.K. (2014). Synthesis of MgO Nanoparticles Using *Aspergillus tubingensis* TFR-3. J. Bionanosci..

[13] Fatimah I. (2018). Biosynthesis and characterization of ZnO nanoparticles using rice bran extract as low-cost templating agent. J. Eng. Sci. Technol..

[14] El-Belely E.F., Farag M.M.S., Said H.A., Amin A.S., Azab E., Gobouri A.A., Fouda A. (2021). Green synthesis of zinc oxide nanoparticles (Zno-nps) using *Arthrospira platensis* (class: Cyanophyceae) and evaluation of their biomedical activities. Nanomaterials.

[15] Lashin I., Fouda A., Gobouri A.A., Azab E., Mohammedsaleh Z.M., Makharita R.R. (2021). Antimicrobial and in vitro cytotoxic efficacy of biogenic silver nanoparticles (Ag-nps) fabricated by callus extract of *Solanum incanum* L.. Biomolecules.

[16] Velgosova O., Dolinská S., Mražíková A., Briančin J. (2020). Effect of *P. kessleri* extracts treatment on AgNPs synthesis. Inorg. Nano-Met. Chem..

[17] Spagnoletti F.N., Spedalieri C., Kronberg F., Giacometti R. (2019). Extracellular biosynthesis of bactericidal Ag/AgCl nanoparticles for crop protection using the fungus *Macrophomina phaseolina*. J. Environ. Manag..

[18] Shafiq T., Uzair M., Iqbal M.J., Zafar M., Hussain S.J., Shah S.A.A. (2021). Green synthesis of metallic nanoparticles and their potential in bio-medical applications. Nano Biomed. Eng..

[19] Fouda A., Abdel-Maksoud G., Saad H.A., Gobouri A.A., Mohammedsaleh Z.M., El-Sadany M.A.H. (2021). The efficacy of silver nitrate (AgNO_3_) as a coating agent to protect paper against high deteriorating microbes. Catalysts.

[20] Fouda A., Awad M.A., Eid A.M., Saied E., Barghoth M.G., Hamza M.F., Awad M.F., Abdelbary S., Hassan S.E.D. (2021). An eco-friendly approach to the control of pathogenic microbes and *Anopheles stephensi* malarial vector using magnesium oxide nanoparticles (Mg-nps) fabricated by *Penicillium chrysogenum*. Int. J. Mol. Sci..

[21] Khalil A.M.A., Hassan S.E.D., Alsharif S.M., Eid A.M., Ewais E.E.D., Azab E., Gobouri A.A., Elkelish A., Fouda A. (2021). Isolation and characterization of fungal endophytes isolated from medicinal plant *Ephedra pachyclada* as plant growth-promoting. Biomolecules.

[22] Rahman A.U., Wei Y., Ahmad A., Khan A.U., Ali R., Ullah S., Ahmad W., Yuan Q. (2020). Selenium Nanorods Decorated Gold Nanostructures: Synthesis, Characterization and Biological Applications. J. Clust. Sci..

[23] Geoffrion L.D., Hesabizadeh T., Medina-Cruz D., Kusper M., Taylor P., Vernet-Crua A., Chen J., Ajo A., Webster T.J., Guisbiers G. (2020). Naked Selenium Nanoparticles for Antibacterial and Anticancer Treatments. ACS Omega.

[24] Filipović N., Ušjak D., Milenković M.T., Zheng K., Liverani L., Boccaccini A.R., Stevanović M.M. (2021). Comparative Study of the Antimicrobial Activity of Selenium Nanoparticles With Different Surface Chemistry and Structure. Front. Bioeng. Biotechnol..

[25] Vahdati M., Tohidi Moghadam T. (2020). Synthesis and Characterization of Selenium Nanoparticles-Lysozyme Nanohybrid System with Synergistic Antibacterial Properties. Sci. Rep..

[26] Liao W., Yu Z., Lin Z., Lei Z., Ning Z., Regenstein J.M., Yang J., Ren J. (2016). Biofunctionalization of Selenium Nanoparticle with *Dictyophora indusiata* Polysaccharide and Its Antiproliferative Activity through Death-Receptor and Mitochondria-Mediated Apoptotic Pathways. Sci. Rep..

[27] Menon S., Agarwal H., Venkat Kumar S., Rajeshkumar S., Shukla A.K., Iravani S. (2019). Chapter 8—Biomemetic synthesis of selenium nanoparticles and its biomedical applications. Green Synthesis, Characterization and Applications of Nanoparticles.

[28] Klaine S.J., Alvarez P.J.J., Batley G.E., Fernandes T.F., Handy R.D., Lyon D.Y., Mahendra S., McLaughlin M.J., Lead J.R. (2008). Nanomaterials in the environment: Behavior, fate, bioavailability, and effects. Environ. Toxicol. Chem..

[29] Pitt J.I., Hocking A.D. (2009). Fungi and Food Spoilage.

[30] White T.J., Bruns T., Lee S., Taylor J. (1990). Amplification and direct sequencing of fungal ribosomal RNA genes for phylogenetics. PCR Protoc. Guide Methods Appl..

[31] Amin M.A., Ismail M.A., Badawy A.A., Awad M.A., Hamza M.F., Awad M.F., Fouda A. (2021). The Potency of Fungal-Fabricated Selenium Nanoparticles to Improve the Growth Performance of *Helianthus annuus* L. and Control of Cutworm *Agrotis ipsilon*. Catalysts.

[32] Vahidi H., Kobarfard F., Kosar Z., Mahjoub M.A., Saravanan M., Barabadi H. (2020). Mycosynthesis and characterization of selenium nanoparticles using standard *Penicillium chrysogenum* PTCC 5031 and their antibacterial activity: A novel approach in microbial nanotechnology. Nanomed. J..

[33] Salem S.S., Fouda M.M.G., Fouda A., Awad M.A., Al-Olayan E.M., Allam A.A., Shaheen T.I. (2021). Antibacterial, Cytotoxicity and Larvicidal Activity of Green Synthesized Selenium Nanoparticles Using *Penicillium corylophilum*. J. Clust. Sci..

[34] Eveland L.K., Haseeb M.A. (2011). Laboratory Rearing of *Biomphalaria glabrata* Snails and Maintenance of Larval Schistosomes In Vivo and In Vitro. Biomphalaria Snails and Larval Trematodes.

[35] WHO (1983). Report of the Scientific Working Group on Plant Molluscicide & Guidelines for Evaluation of Plant Molluscicides.

[36] WHO (1965). Molluscicide screening and evaluation. Bull. World Health Organ..

[37] Hamdi S.A.H., Ibrahim A.M., Ghareeb M.A., Fol M.F. (2021). Chemical characterization, biocidal and molluscicidal activities of chitosan extracted from the crawfish *Procambarus clarkii* (Crustacea: Cambaridae). Egypt. J. Aquat. Biol. Fish..

[38] Eissa M.M., El Bardicy S., Tadros M. (2011). Bioactivity of miltefosine against aquatic stages of *Schistosoma mansoni*, *Schistosoma haematobium* and their snail hosts, supported by scanning electron microscopy. Parasit. Vectors.

[39] Nduku W.K., Harrison A.D. (1980). Cationic responses of organs and haemolymph of *Biomphalaria pfeifferi* (Krauss), *Biomphalaria glabrata* (Say) and *Helisoma trivolvis* (Say)(Gastropoda: Planorbirdae) to cationic alterations of the medium. Hydrobiologia.

[40] Abdul-Salam J.M., Michelson E.H. (1980). *Biomphalaria glabrata* amoebocytes: Effect of *Schistosoma mansoni* infection on in vitro phagocytosis. J. Invertebr. Pathol..

[41] Grazeffe V.S., de Freitas Tallarico L., de Sa Pinheiro A., Kawano T., Suzuki M.F., Okazaki K., de Bragança Pereira C.A., Nakano E. (2008). Establishment of the comet assay in the freshwater snail *Biomphalaria glabrata* (Say, 1818). Mutat. Res.—Genet. Toxicol. Environ. Mutagen..

[42] Ibrahim A.M., Sayed D.A. (2019). Toxicological impact of oxyfluorfen 24% herbicide on the reproductive system, antioxidant enzymes, and endocrine disruption of *Biomphalaria alexandrina* (Ehrenberg, 1831) snails. Environ. Sci. Pollut. Res. Int..

[43] Aebi H. (1984). Catalase in vitro. Methods Enzymol..

[44] Ohkawa H., Ohishi N., Yagi K. (1979). Assay for lipid peroxides in animal tissues by thiobarbituric acid reaction. Anal. Biochem..

[45] Beutler E., Duron O., Kelly B.M. (1963). Improved method for the determination of blood glutathione. J. Lab. Clin. Med..

[46] Koracevic D., Koracevic G., Djordjevic V., Andrejevic S., Cosic V. (2001). Method for the measurement of antioxidant activity in human fluids. J. Clin. Pathol..

[47] Bellos J.K., Perrea D.N., Theodotopoulou E., Vlachos I., Kostakis A.I. (2011). Nitric oxide production as an indicator of recurrence of focal and segmental glomerulosclerosis following kidney transplantation. Saudi J. Kidney Dis. Transpl..

[48] Finney D.J. (1971). Probit Analysis.

[49] Murray R. (1981). Speigel: Statistical estimation theory. Schaum’s Outline Series: Theory and Problems of Statistics in SI Units.

[50] Oyeyemi O.T. (2021). Application of nanotized formulation in the control of snail intermediate hosts of schistosomes. Acta Trop..

[51] Pohl C., Polli F., Schütze T., Viggiano A., Mózsik L., Jung S., de Vries M., Bovenberg R.A.L., Meyer V., Driessen A.J.M. (2020). A *Penicillium rubens* platform strain for secondary metabolite production. Sci. Rep..

[52] Joshi S.M., De Britto S., Jogaiah S., Ito S.I. (2019). Mycogenic selenium nanoparticles as potential new generation broad spectrum antifungal molecules. Biomolecules.

[53] Ramamurthy C.H., Sampath K.S., Arunkumar P., Kumar M.S., Sujatha V., Premkumar K., Thirunavukkarasu C. (2013). Green synthesis and characterization of selenium nanoparticles and its augmented cytotoxicity with doxorubicin on cancer cells. Bioprocess Biosyst. Eng..

[54] Worrall E.A., Hamid A., Mody K.T., Mitter N., Pappu H.R. (2018). Nanotechnology for plant disease management. Agronomy.

[55] Zare B., Babaie S., Setayesh N., Shahverdi A.R., Shahverdi A. (2013). Isolation and characterization of a fungus for extracellular synthesis of small selenium nanoparticles Extracellular synthesis of selenium nanoparticles using fungi. Nanomed. J..

[56] Ahmad M.S., Yasser M.M., Sholkamy E.N., Ali A.M., Mehanni M.M. (2015). Anticancer activity of biostabilized selenium nanorods synthesized by *Streptomyces bikiniensis* strain Ess_amA-1. Int. J. Nanomed..

[57] Ullah A., Yin X., Wang F., Xu B., Mirani Z.A., Xu B., Chan M.W.H., Ali A., Usman M., Ali N. (2021). Biosynthesis of selenium nanoparticles (via *Bacillus subtilis* bsn313), and their isolation, characterization, and bioactivities. Molecules.

[58] Ranjitha V.R., Ravishankar V.R. (2018). Extracellular Synthesis of Selenium Nanoparticles from an Actinomycetes *Streptomyces griseoruber* and Evaluation of its Cytotoxicity on HT-29 Cell Line. Pharm. Nanotechnol..

[59] Salem S.S. (2022). Bio-fabrication of Selenium Nanoparticles Using Baker’s Yeast Extract and Its Antimicrobial Efficacy on Food Borne Pathogens. Appl. Biochem. Biotechnol..

[60] Barabadi H., Kobarfard F., Vahidi H. (2018). Biosynthesis and Characterization of Biogenic Tellurium Nanoparticles by Using *Penicillium chrysogenum* PTCC 5031: A Novel Approach in Gold Biotechnology. Iran. J. Pharm. Res. IJPR.

[61] Al-Brakati A., Alsharif K.F., Alzahrani K.J., Kabrah S., Al-Amer O., Oyouni A.A., Habotta O.A., Lokman M.S., Bauomy A.A., Kassab R.B. (2021). Using green biosynthesized lycopene-coated selenium nanoparticles to rescue renal damage in glycerol-induced acute kidney injury in rats. Int. J. Nanomed..

[62] Rajkumar K., Sandhya M.V.S., Koganti S., Burgula S. (2020). Selenium nanoparticles synthesized using *Pseudomonas stutzeri* (Mh191156) show antiproliferative and anti-angiogenic activity against cervical cancer cells. Int. J. Nanomed..

[63] Alagesan V., Venugopal S. (2019). Green Synthesis of Selenium Nanoparticle Using Leaves Extract of *Withania somnifera* and Its Biological Applications and Photocatalytic Activities. Bionanoscience.

[64] Lian S., Diko C.S., Yan Y., Li Z., Zhang H., Ma Q., Qu Y. (2019). Characterization of biogenic selenium nanoparticles derived from cell-free extracts of a novel yeast *Magnusiomyces ingens*. 3 Biotech.

[65] Osman G.Y., Ahmed M.M., Abdel Kader A., Mohamed A.A. (2013). Biological and biochemical impacts of the fungal extract of *Aspergillus fumigatus* on *Biomphalaria alexandrina* snails infected with *Schistosoma mansoni*. Biosciences.

[66] Mahmoudvand H., Shakibaie M., Tavakoli R., Jahanbakhsh S., Sharifi I. (2014). In Vitro Study of Leishmanicidal Activity of Biogenic Selenium Nanoparticles against Iranian Isolate of Sensitive and Glucantime-Resistant *Leishmania tropica*. Iran. J. Parasitol..

[67] Alkhudhayri A., Al-Shaebi E.M., Qasem M.A.A., Murshed M., Mares M.M., Al-Quraishy S., Dkhil M.A. (2020). Antioxidant and anti-apoptotic effects of selenium nanoparticles against murine eimeriosis. An. Acad. Bras. Cienc..

[68] Dkhil M.A., Khalil M.F., Diab M.S.M., Bauomy A.A., Santourlidis S., Al-Shaebi E.M., Al-Quraishy S. (2019). Evaluation of nanoselenium and nanogold activities against murine intestinal schistosomiasis. Saudi J. Biol. Sci..

[69] De Carvalho Augusto R., Merad N., Rognon A., Gourbal B., Bertrand C., Djabou N., Duval D. (2020). Molluscicidal and parasiticidal activities of *Eryngium triquetrum* essential oil on *Schistosoma mansoni* and its intermediate snail host *Biomphalaria glabrata*, a double impact. Parasites Vectors.

[70] Ibrahim A.M., Ahmed A.K., Bakry F.A., Abdel-Ghaffar F. (2018). Hematological, physiological and genotoxicological effects of Match 5% EC insecticide on *Biomphalaria alexandrina* snails. Ecotoxicol. Environ. Saf..

[71] Ali D. (2014). Evaluation of environmental stress by comet assay on freshwater snail *Lymnea luteola* L. exposed to titanium dioxide nanoparticles. Toxicol. Environ. Chem..

[72] Ibrahim A.M., Ghoname S.I. (2018). Molluscicidal impacts of *Anagallis arvensis* aqueous extract on biological, hormonal, histological and molecular aspects of *Biomphalaria alexandrina* snails. Exp. Parasitol..

[73] Wang Z., Zhang L., Zhao J., Xing B. (2016). Environmental processes and toxicity of metallic nanoparticles in aquatic systems as affected by natural organic matter. Environ. Sci. Nano.

[74] Prokhorova E.E., Serebryakova M.K., Tokmakova A.S., Ataev G.L. (2018). Hemocytes of mollusc *Biomphalaria glabrata* (Gastropoda, pulmonata). Invertebr. Surviv. J..

[75] Cavalcanti M.G.S., Filho F.C., Mendonça A.M.B., Duarte G.R., Barbosa C.C.G.S., De Castro C.M.M.B., Alves L.C., Brayner F.A. (2012). Morphological characterization of hemocytes from *Biomphalaria glabrata* and *Biomphalaria straminea*. Micron.

[76] Abdel-Hamid H., Mekawey A.A.I. (2014). Biological and hematological responses of *Biomphalaria alexandrina* to mycobiosynthsis silver nanoparticles. J. Egypt. Soc. Parasitol..

[77] Ibrahim A.M., Ahmed A.K., Bakry F.A., Rabei I., Abdel-Ghaffar F. (2019). Toxicological impact of butralin, glyphosate-isopropylammonium and pendimethalin herbicides on physiological parameters of *Biomphalaria alexandrina* snails. Molluscan Res..

[78] Ray M., Bhunia N.S., Bhunia A.S., Ray S. (2013). A comparative analyses of morphological variations, phagocytosis and generation of cytotoxic agents in flow cytometrically isolated hemocytes of Indian molluscs. Fish Shellfish Immunol..

[79] Donaghy L., Hong H.-K., Lambert C., Park H.-S., Shim W.J., Choi K.-S. (2010). First characterisation of the populations and immune-related activities of hemocytes from two edible gastropod species, the disk abalone, *Haliotis discus discus* and the spiny top shell, *Turbo cornutus*. Fish Shellfish Immunol..

[80] Khalil A.M. (2015). Toxicological effects and oxidative stress responses in freshwater snail, *Lanistes carinatus*, following exposure to chlorpyrifos. Ecotoxicol. Environ. Saf..

[81] Barky F.A., Abdelsalam H.A., Mahmoud M.B., Hamdi S.A.H. (2012). Influence of Atrazine and Roundup pesticides on biochemical and molecular aspects of *Biomphalaria alexandrina* snails. Pestic. Biochem. Physiol..

[82] Sayed S.S.M., Abdel-Wareth M.T.A. (2017). The comparative effect of chlorine and Huwa-san as disinfecting agents on *Biomphalaria alexandrina* snails and free larval stages of *Schistosoma mansoni*. Parasitol. Res..

[83] Wang W., Mao Q., Yao J., Yang W., Zhang Q., Lu W., Deng Z., Duan L. (2018). Discovery of the pyridylphenylureas as novel molluscicides against the invasive snail *Biomphalaria straminea*, intermediate host of *Schistosoma mansoni*. Parasites Vectors.

[84] Saleh H.A., Abdel-Motleb A., Habib M.R. (2021). Neurotoxicity and genotoxicity of the veterinary antibiotics oxytetracycline and trimethoprim-sulphadiazine to *Biomphalaria alexandrina* snails. Int. J. Environ. Stud..

[85] Chemical Computing Group Inc (2016). Molecular operating environment (MOE). https://www.chemcomp.com/Products.htm.

[86] Manjula R., Wright G.S.A., Strange R.W., Padmanabhan B. (2018). Assessment of ligand binding at a site relevant to SOD1 oxidation and aggregation. FEBS Lett..

[87] Oakley A.J., Lo Bello M., Nuccetelli M., Mazzetti A.P., Parker M.W. (1999). The ligandin (non-substrate) binding site of human Pi class glutathione transferase is located in the electrophile binding site (H-site). J. Mol. Biol..

[88] Kim G.H., Kim J.E., Rhie S.J., Yoon S. (2015). The Role of Oxidative Stress in Neurodegenerative Diseases. Exp. Neurobiol..

[89] Bastos F.F., Tobar S.A.L., Dantas R.F., Silva E.S., Nogueira N.P.A., Paes M.C., Righi B.D.P., Bastos J.C., Bastos V.L.F.C. (2013). Melatonin affects conjugation of 4-hydroxynonenal with glutathione in liver of pacu, a hypoxia-tolerant fish. Fish Physiol. Biochem..

[90] Montassir L., Berrebaan I., Mellouki F., Zkhiri F., Boughribil S., Bessi H. (2017). Acute toxicity and reprotoxicity of aqueous extract of a Moroccan plant (*Tetraclinis articulata*) on freshwater cladoceran *Daphnia magna*. J. Mater. Environ. Sci..

